# The present and future of blended care: current research and introduction to the B-FIT framework

**DOI:** 10.1038/s41746-026-02526-4

**Published:** 2026-03-26

**Authors:** Laura Luisa Bielinski, Rebekka Büscher, Severin Hennemann, Carmen Henning, Simon H. Kohl, Caroline Meyer, Annika S. Reinhold, Lena Sophia Steubl, Ingrid Titzler, Lea Vogel, Anna-Carlotta Zarski, Carmen Schaeuffele

**Affiliations:** 1 Department of Clinical Psychology and Psychotherapy, University of Bern, Bern, Switzerland; 2 Institute of Medical Psychology and Medical Sociology, Medical Faculty, University of Freiburg, Freiburg, Germany; 3 Department of Clinical Psychology, Psychotherapy and Experimental Psychopathology, Johannes Gutenberg-University Mainz, Mainz, Germany; 4 Professorship of Psychopathology, Institute of Psychology, University of Bamberg, Bamberg, Germany; 5 Department of Child and Adolescent Psychiatry, Psychosomatics and Psychotherapy, Faculty of Medicine, RWTH Aachen University, Aachen, Germany; 6 JARA-Institute Molecular Neuroscience and Neuroimaging (INM-11), Forschungszentrum Jülich, Jülich, Germany; 7 Clinical Psychology and Psychotherapy, Dept. of Education and Psychology, Freie Universität Berlin, Berlin, Germany; 8 Department of Public Mental Health, Central Institute of Mental Health, Medical Faculty Mannheim, University of Heidelberg, Mannheim, Germany; 9 German Center for Mental Health (DZPG), partner site, Mannheim-Heidelberg-Ulm, Heidelberg, Germany; 10 Department of Clinical Psychology and Psychotherapy, Ulm University, Ulm, Germany; 11 Department of Clinical Psychology and Psychotherapy, Institute of Psychology, University of Erlangen-Nürnberg, Erlangen, Germany; 12 PRIVATKLINIK REGENA Bad Brückenau, Bad Brückenau, Germany; 13 Department of Psychology, LMU Munich, Munich, Germany; 14 German Center for Mental Health (DZPG), partner site, Munich, Germany; 15 Division of eHealth in Clinical Psychology, Department of Clinical Psychology, Philipps Universiät Marburg, Marburg, Germany; 16 Clinical-Psychological Intervention, Dept. of Education and Psychology, Freie Universität Berlin, Berlin, Germany

**Keywords:** Psychiatric disorders, Health care

## Abstract

Blended care (BC), combining face-to-face therapy with digital components, is gaining momentum in the field of mental health, yet lacks conceptual clarity. This perspective paper outlines a dimensional conceptualization of BC and introduces the B-FIT (Blend-Focus-Integration-Timing) framework. We highlight the need to refine the theoretical foundations of BC, strengthen the evidence base for its effectiveness, and integrate stakeholder perspectives to inform future research and support the successful implementation of BC.

## The present and future of blended care: current research and introduction to the B-FIT framework

Mental disorders represent one of the pressing public health challenges of our time^1,2^. Despite the availability of evidence-based interventions, significant treatment gaps underscore the need for innovative approaches. One promising avenue is the combination of face-to-face therapy with digital components. Digital components could potentially enhance the effectiveness and efficiency of traditional therapy and alleviate pressure on overburdened mental health systems. Face-to-face therapy components can provide the personal element often lacking in stand-alone digital interventions and may enhance engagement. To leverage these advantages, digital and face-to-face components (“blended care”, BC) can be combined in different ways. This perspective paper has several aims: First, we highlight how blended care is currently conceptualized in the literature, then we narratively review the empirical evidence regarding its efficacy and efficiency as well as moderators/mediators, before highlighting implementation barriers and facilitators. To move the field forward, we propose a novel dimensional conceptualization of BC by introducing the B-FIT (Blend-Focus-Integration-Timing) framework and outlining future directions for research and practice.

## Current conceptualizations of blended care

The combination of face-to-face therapy and digital components in the specific context of mental health has been conceptualized in a variety of ways^3–9^. This is reflected in the diverse terminology populating the literature, for example, BC, blended therapy, or hybrid therapy. In this perspective paper, we will use the term BC to refer to all combinations of face-to-face therapy and digital components for mental health treatment.

Viewed broadly, BC can be examined by looking at different levels of treatment^4^. On a macro level, i.e., the overall treatment journey that a patient undertakes, the digital component can be used intentionally before, during, or after a course of face-to-face therapy. This would also include what Erbe et al.^6^ considered sequential blends where a digital intervention is provided before (“stepping up” to bridge waiting times or as part of a stepped care approach) or after (“stepping down” as relapse prevention or aftercare) treatment. At the meso level—during a course of face-to-face therapy—digital components might, for example, be used alongside face-to-face therapy (add-on blends) or be integrated within face-to-face therapy (integrating blends), implying a higher degree of interconnection between digital and face-to-face components. On a micro level, ways in which digital components can be combined with face-to-face therapy within a single therapy session are examined. Macro, meso, and micro levels are used conceptually to describe levels of blending within the treatment process rather than in a systems-theory sense. At every level of granularity, dimensions shaping BC design, implementation, and evaluation as specified by Chen et al.^5^ can be considered. These include intervention components (telehealth, AR/VR, app/computer, chatbot, in-person), the clinical target (serious mental illness, anxiety/mood, other), and human support (frequency, intensity).

## An overview of current research on BC

### Efficacy, effectiveness, and efficiency of BC

A growing body of literature explores the potential of BC models across diverse clinical contexts and populations^6,7,10–12^. However, studies included in these reviews are highly heterogeneous in terms of designs and outcome measures, and BC is often compared to treatment-as-usual (e.g., treatment by GP, case management, support groups, etc.), not only to psychotherapy. In addition, several BC studies have been underpowered. Noninferiority studies on BC typically have involved fewer than 200 participants and used noninferiority margins that allow for small to moderate differences between treatments^13–16^.

In pursuit of enhanced efficacy and effectiveness of therapy, some studies have demonstrated positive effects of BC, albeit predominantly for depression and anxiety^6,17–19^. Notably, emerging, albeit heterogeneous, evidence suggests that the benefits of BC may extend beyond these disorders and symptom groups. For example, in the context of addiction, Carroll et al.^20^ found that patients receiving a computer-based intervention in addition to standard treatment had longer periods of abstinence. BC has also found applications in patients with psychosis, as highlighted by a recent review^10^: Out of 11 studies, 87% identified improvements in symptomatology and recovery parameters. BC is of course not exclusive to adult populations: The successful application of “Moderated Online Social Therapy” (MOST) that blends human and digital support for youth populations has been detailed in a recent publication by Alvarez-Jimenz et al.^21^. Complementing these findings, studies report that there is an added effect attributable to apps as adjuncts for the treatment of a variety of mental health disorders^22^. Notably, a recent large-scale study in which psychotherapy combined with transdiagnostic online modules was compared to psychotherapy alone did not find increased effectiveness—potentially due to implementation barriers in routine care, including limited use of the digital intervention^23^.

Given the heterogeneous and still inconclusive evidence regarding BC’s potential to increase effectiveness, many BC initiatives are motivated less by expectations of superior clinical outcomes and more by the pursuit of greater efficiency. A central goal of BC is to achieve comparable outcomes with fewer resources and enable greater flexibility and scalability in service delivery through digital components. Although evidence quality remains variable, there are studies providing indications that therapist time may be reduced in BC while maintaining effects, e.g., in patients with depression^14–16,24–26^, anxiety^25,27^, or post-traumatic stress disorder^13^. Findings on the cost-effectiveness of BC vary depending on societal or healthcare provider perspective^26–29^, therefore no clear verdict on BC’s cost-effectiveness has yet been reached.

Lengthy waiting times and insufficient post-treatment support remain persistent challenges in mental health care^30,31^. Consequently, integrating digital tools before and after therapy represents important applications of BC. Thus, the integration of digital tools in periods before and after therapy plays an important role. Krämer et al.^32^ demonstrated that a digital intervention provided to patients on a waitlist for psychotherapy outperformed treatment-as-usual regarding depressive symptom reduction. However, a more recent systematic review by Huang et al.^33^ reported that digital interventions used while waiting may not be more effective compared to simply waiting or using a self-help book. Posttreatment, digital tools have shown promise in sustaining therapeutic gains and preventing relapse, particularly for depression and anxiety^34,35^. Nonetheless, the extent to which these interventions are explicitly designed and evaluated for follow-up care after psychotherapy remains limited. In sum, it would be of value to examine how blends might increase seamless continuity of care through the entire arc of a patient’s treatment journey.

### Moderators and mechanisms of BC: understanding who benefits and why

Evidence on who benefits most from BC remains limited. According to Fenski et al.^36^, there are no demographic characteristics that currently make the use of BC more or less advisable, but a patient’s personal preference seems to matter. One study was able to show that adherence is higher among those who prefer BC, are more educated, and have fewer comorbidities^37^. Another study showed that participants were significantly more likely to drop out if they did not complete the digital activities assigned early in treatment, were female, reported more severe depressive symptoms at baseline, reported taking antidepressants, and did not disclose their ethnicity^38^. Given the preference of the younger generation of digital natives for online treatments and BC approaches^39,40^, it can be assumed that they might benefit particularly from the integration of digital components into psychotherapy. In youth, digital interventions often include more therapist support than adult-targeted ones, though comparative efficacy data remain sparse^41,42^. Supported formats generally show greater adherence and effectiveness than fully automated ones^43^. The therapeutic alliance, a well-studied mechanism in traditional therapy, has shown mixed findings in BC. Some studies suggest it may matter less than in traditional therapy^44^, others that it may even be enhanced^45^. Finally, the structure of how both elements are integrated seems to matter: six or fewer face-to-face sessions (vs. more than six), and more than 50% of sessions delivered digitally (vs. >50% delivered face-to-face) were linked to better outcomes for both depression and anxiety^7^.

### Barriers and facilitators for implementation of BC

The implementation of BC in routine care appears to be associated with barriers and facilitators at various levels. From the healthcare professionals’ perspective, BC provides a better opportunity to establish rapport than self-guided treatment and allows active monitoring and follow-up of patients as they work through online elements^46^. BC empowers patients’ self-management and autonomy and can enhance the transfer of therapeutic content into daily life^8,11,47,48^, which may reinforce patients and therapists to use BC.

Barriers to implementation include limited access to patients’ digital treatment progress^49^, technical issues, and a lack of information on BC^47^. While BC may reduce clinician time^6^, therapists emphasize that additional effort or workload such as getting familiar with digital components must be considered^46,50–52^.

Shifting to the patient’s perspective, advantages of BC include digital and face-to-face elements transforming and complementing each other^49^ and patients being able to discuss online content face-to-face with a therapist^53^. In a similar vein, participants are motivated to persist with BC when their overall need for relatedness is satisfied, for example, through connectedness with a therapist^52^. Barriers include problems with technology^53^, face-to-face and digital components not being integrated enough^49^, and patients lacking energy and motivation to meet both the requirements of digital and face-to-face components^53^.

Regarding children and adolescents, implementation can be facilitated or hindered by parents or caregivers, who act as gatekeepers and guides in the use of digital components. Ideally, parents/caregivers are integrated into the therapeutic process and support their child in engaging with digital tools, especially for children who lack the motivational and self-regulation resources^54^. However, digital elements may also cause tension, particularly if perceived as controlling or if they disrupt routines and screen time^54^. Another potential barrier is that digital therapeutic content must compete with digital leisure activities like gaming and social media^54^. Therefore, digital components must be carefully designed to align with the developmental needs and media habits of the specific target population^54–56^.

**Fig. 1:**
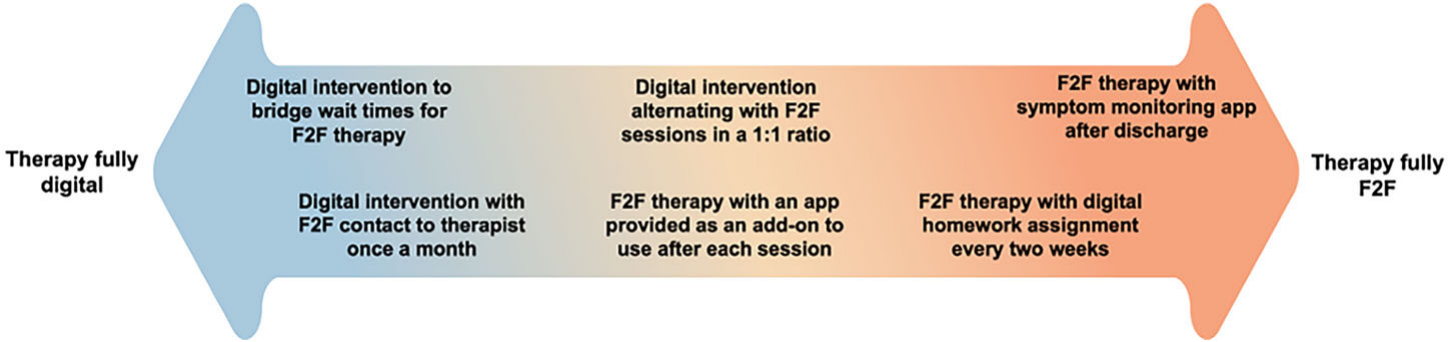
Blended care as a continuum between fully digital and fully face-to-face therapy. This figure illustrates the conceptual range of blended care (BC), which combines digital and face-to-face (F2F) therapy elements. BC occupies the space between fully digital and fully F2F care models, without representing either extreme. The blue-to-red gradient represents the transition from digital-dominant to F2F-dominant approaches. Displayed examples are prototypical formats along this continuum. On the digital-dominant end are interventions such as digital programs used during waiting periods for therapy, or digital tools with minimal therapist contact (e.g., monthly check-ins). In the middle are balanced formats like alternating digital and F2F sessions or F2F sessions linked with digital components after each encounter. On the F2F-dominant end are formats like in-person therapy with symptom monitoring apps post-discharge or biweekly digital homework supplements. These examples represent only a subset of possible configurations. In practice, BC allows for diverse and flexible combinations of digital and F2F components, which may serve different functions across treatment stages.

To illustrate the practical experiences related to the implementation of BC, we asked researchers and practitioners to share their main learnings from implementing BC using recruitment via a snowball system (see Supplementary Material for detailed methods and results). Lessons learned are summarized in Fig. 1.

## Introducing the dimensional B-FIT model for planning and evaluating BC

**Fig. 2:**
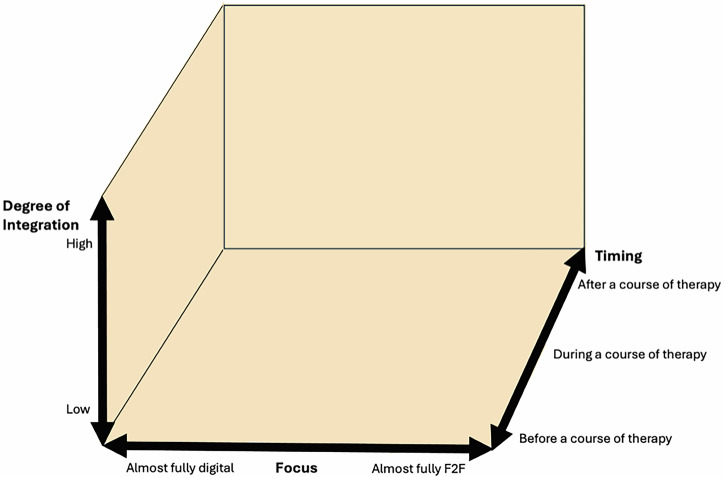
The B-FIT framework for conceptualizing blended care. This figure presents the B-FIT framework, a three-dimensional model that conceptualizes blended care (BC) along continuous axes of focus, degree of integration, and timing. Axes are depicted in solid black to delineate the three conceptual dimensions. The background is shaded light orange to highlight the open space in which various blended care formats can be positioned. Focus (horizontal axis) describes the relative emphasis of digital versus face-to-face (F2F) components within a blended care format. It ranges from digital-dominant at one end to F2F dominant on the other. Degree of integration (vertical axis) captures how closely digital and F2F components are coordinated, from low (minimal connection between components) to high (components are interdependent, interwoven and designed to function together). In the figure, the timing dimension represents macro-temporal placement of digital components relative to F2F therapy (before, during, or after). Digital components may also be orchestrated more finely within these macro positions, for example, occurring between sessions, aligned with session phases, or delivered just-in-time in response to user states or questionnaire inputs. These fine-grained timing strategies are nested within the macro-temporal positions.

As revealed by our narrative review of BC’s effectiveness, efficiency, moderators/mechanisms and implementation challenges, the evidence remains fragmented and inconclusive. Since existing studies often use the term heterogeneously, grouping together highly divergent interventions under the “BC” umbrella, the evidence base is difficult to compare or synthesize. To address this conceptual gap, we propose to view BC as a dimensional concept that occupies the space between fully digital and fully face-to-face but excludes both extremes. This dimensional perspective fills a gap in the literature, namely the lack of a consistent conceptual framework for defining what constitutes BC. Figure 2 shows examples of blends that can be situated on this spectrum. The examples are not an exhaustive list but are meant to provide prototypes. To conceptualize BC as dimensional aligns with a broader movement in psychology and mental health toward dimensional conceptualizations, as exemplified by initiatives such as Research Domain Criteria^57^ and Hierarchical Taxonomy of Psychopathology^58^. Although these frameworks are far more complex and operate at different levels of analysis, they demonstrate the value of organizing complex phenomena along graded or hierarchical dimensions rather than discrete categories.

**Fig. 3:**
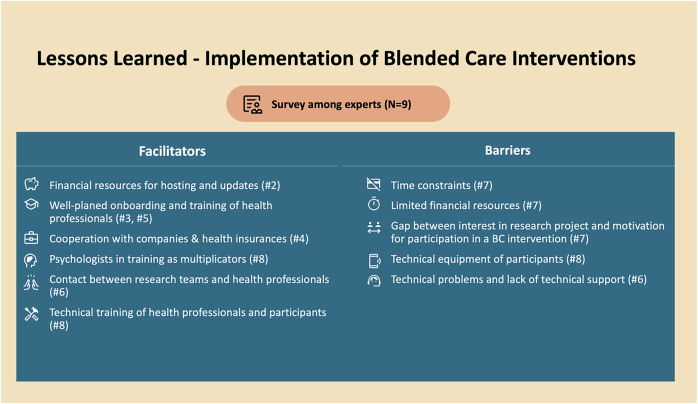
Facilitators and barriers to the implementation of BC were mentioned by researchers and practitioners. *Note*. This figure summarizes key facilitators and barriers identified in the implementation of blended care (BC) interventions, based on an expert survey (*N* = 9). The left column presents factors that support successful implementation, while the right column highlights common barriers.

Within the BC spectrum, the B-FIT framework (Fig. 3) characterizes any given blend by the dimensions (1) modality **f**ocus (how much of what?), (2) degree of **i**ntegration (how interwoven?), and (3) **t**iming (when?). This enables a more precise conceptualization of BC and facilitates treatment planning and evaluation. The dimension modality focus at its broadest level reflects the overall emphasis of the blend, whether a blend leans more towards therapeutic content delivered face-to-face, digitally, or relatively equally by both modalities. This corresponds to Ferrao Nunes-Zlotokowski et al.^7^, who distinguish between core and supplementary components, with core components representing the essential parts of a blended configuration. Viewed more finely, the same dimension also captures how well specific elements of a blend align with a particular modality. Here, modality focus is also about what types of activities or content are best delivered through each modality. Media Richness Theory^59^ can be a useful lens: different media vary in their effectiveness for conveying complex or ambiguous information, and this can inform which elements are better situated in digital versus face-to-face formats.

Degree of integration can be understood as a dimension that characterizes how tightly the digital and face-to-face components of a blended therapy configuration are interconnected. This dimension reflects the extent to which digital and in-person elements are woven together. Conceptually, this notion of integration is consistent with principles from sociotechnical systems theory, which focuses on the coordinated design and joint optimization of human and technological components within complex systems^60^.

Timing can be understood as a dimension that characterizes when a digital component is positioned relative to a face-to-face component. At a macro level, this refers to whether a digital component is introduced before, during, or after the course of face-to-face treatment. Within these broad temporal regions, timing can also take more fine-grained forms. For example, a digital activity may be placed between sessions, embedded at the start of a session to prime engagement, or delivered immediately afterward to consolidate learning. These more fine-grained temporal variations do not represent separate positions on the timing dimension but rather refinements within a macro placement. For example, placing digital interventions between sessions during a course of face-to-face therapy may align with findings that between-session homework is an active ingredient of therapy^61^.

All three B-FIT dimensions serve a dual purpose: they provide researchers with a systematic way to classify and compare BC setups and studies, while also offering practitioners a practical scaffolding for planning and personalizing their BC configuration. The modality focus dimension invites researchers to describe ratios and emphases of modalities, while encouraging practitioners to reflect on the balance between face-to-face and digital components and on which aspects of therapy are best supported by which modality. The integration dimension allows researchers to distinguish more precisely between BC designs that combine modalities in parallel and those that integrate them more into an interdependent and intertwined process. For practitioners, it serves as a prompt to consider the intended degree of integration: When digital components should be closely linked to face-to-face work to shape the therapeutic process, and when a more independent, adjunctive use of digital tools may better support clinical aims. The timing dimension provides researchers with a systematic way to describe when digital and face-to-face components are combined. For practitioners, it highlights how the temporal placement of digital and in-person elements can best support clinical goals. To illustrate how the B-FIT model can support clinicians, a case example is provided in the supplementary material.

## Future directions

BC has attracted substantial research interest. However, its potential is undermined by a lack of conceptual clarity. To realize the potential of BC, research should focus on (1) a clearer, dimensional conceptualization of BC, with the goal to enhance (2) effectiveness, and (3) implementation.

Currently, the field is dominated by inconsistent terminology and the tendency to lump different combinations of digital and face-to-face elements under the same umbrella term, without adequately describing blend characteristics. Going forward, our recommendations are three-fold. First, we propose to explicitly differentiate between BC as the full spectrum of blend possibilities and a specific subgroup of BC, “blended therapy”, in which both the face-to-face and digital components fulfill a therapeutic function. In contrast to blended therapy, BC also entails setups in which, for example, face-to-face therapy is complemented by a digital component that does not serve therapeutic functions per se, like digital assessment tools or wearables^62^. Second, most trials to date have focused on blends on macro and meso levels, and we would encourage researchers to also initiate research on blends that occur on the session micro level. Such efforts may call for creating blended interventions that are purpose-built for BC from the very beginning, rather than assembling blends from digital tools that were initially intended for self-help. Third, to advance the field, researchers should systematically report not only on the digital and face-to-face components but also on blend characteristics, as captured by the B-FIT framework: focus, integration, and timing. An overview of how a selection of aforementioned randomized controlled trials fit into the B-FIT framework is presented in the supplementary material.

Until blend characteristics are more consistently defined and reported, the understanding of the effectiveness of BC remains limited. Key questions remain: What ratio of digital and face-to-face elements yields optimal outcomes? Which therapeutic tasks are best delivered in which modality? For example, therapists may prefer a 75% face-to-face ratio, while patients lean toward a more balanced 50–60%^8^. Theoretical guidance from Media Richness Theory^59^ supports aligning richer communication media with more complex therapeutic content. Traditionally, digital components are assigned as standardized tasks, while in-person sessions allow for responsive, individualized care. However, emerging technologies, particularly in artificial intelligence (AI), may disrupt these boundaries, enabling more dynamic and personalized digital interventions. First applications of conversational AI homework chatbots, in addition to group psychotherapy, show promising results in engaging patients more in therapy and improving response rates^63^.

Furthermore, the mechanisms of action in BC—and specifically blended therapy—remain underexplored. For example, little attention has been given to whether the combination of digital and face-to-face elements might foster a unique mechanism of action concerning the therapeutic alliance. Research on digital mental health interventions shows that patients can form meaningful alliances even when contact occurs entirely online^64^. Yet rather than asking whether a “digital alliance” is simply equivalent to a traditional one, it may be more productive to consider how it differs^65^. This question is especially relevant in BC, where three elements interact: the digital element, the therapist, and the patient. In BC, the therapeutic alliance may function differently than in either face-to-face or solely digital therapy because it is co-constructed across multiple modalities. Whereas face-to-face therapy relies primarily on in-session interpersonal processes and digital-only formats depend on mediated communication, BC distributes alliance formation across both human and digital interactions. Building on Bordin’s^66^ conceptualization of the alliance as comprising agreement on goals, collaboration on tasks, and the emotional bond, BC may extend and reshape how these components operate. Early findings on alliance in BC suggest that although human support remains meaningful, digital elements can promote autonomy and self-directed engagement, potentially strengthening goal agreement and task collaboration^67^. At the same time, digital components may carry a symbolic sense of the therapist’s presence into daily life, extending the relational connection beyond the therapy room. In this way, the BC alliance becomes partially asynchronous, technologically mediated, and potentially more continuous, suggesting that BC may not merely replicate traditional alliance processes but introduces a hybrid form of connection. Identifying such potential mechanisms, along with potentially meaningful moderators, is essential to determine for whom and under what conditions BC is most effective.

As BC evolves, its potential drawbacks must also be examined in more detail. While BC has been suggested to reduce dropout rates, there is currently no consensus on how dropout is defined in the context of BC, as patients could drop out of either component^68^. Importantly, combining two treatments may increase the opportunities for disengagement rather than reduce them. These considerations highlight that questions of effectiveness cannot be separated from how BC is implemented in real-world settings.

Implementation of BC is still at an early stage. Next steps should include synthesizing barriers and facilitators of BC across stakeholder groups, using participatory methods to ensure user-centered design (e.g., WHO’s framework for meaningful engagement^69^), and attending to organizational and systemic factors outlined in implementation frameworks, such as the Consolidated Framework for Implementation Research^70^. To further guide implementation efforts, the B-FIT framework offers a structure by providing clear parameters: focus, integration, and timing.

While BC is often implemented as a fixed protocol, B-FIT emphasizes flexibility: blend parameters can be continuously adjusted based on patient needs, treatment stage, and clinical goals. Digital platforms that integrate assessment, real-time monitoring, and decision support could further support clinicians in making such adaptive adjustments. Along these lines, B-FIT could be used to examine how certain locations within the dimensional cube may influence clinical outcomes through potential mechanisms. For instance, consider a blend that is face-to-face-dominant, with a low degree of integration, delivered during therapy with digital elements provided between sessions. This specific configuration may have been chosen based on the assumptions that the patient may benefit from (1) richer interpersonal cues, given the emotionally nuanced and ambiguous tasks involved (Media Richness Theory) (focus: face-to-face dominant), (2) reserving in-person time for experiential work while reducing cognitive load during sessions and acknowledging the patient’s high autonomy by the patient working on digital content independently (integration: low), and (3) engaging in digital work alongside therapy to help maintain continuity, reduce skill decay, and support strategy use in daily life^61^ (timing: during therapy; digital between sessions). This example illustrates how B-FIT can be used to generate testable hypotheses about which blended configurations may be most effective for particular clinical scenarios. A concrete example of how a clinician may want to use B-FIT for implementation is presented in the supplementary material.

Looking further ahead, the interaction between the B-FIT dimensions in clinical practice may reveal patterns characteristic of complex adaptive systems. In real clinical settings, the three dimensions are unlikely to function in isolation: small shifts in one may produce non-linear changes in others. Understanding such emergent configurations represents a critical frontier for future research and for realizing the full potential of BC.

## Conclusion

A dimensional conceptualization of BC opens new directions for research and practice. BC is not a panacea, but when guided by a clear conceptual framework and informed by real-world implementation practices, it offers a pathway for reshaping mental health treatment to potentially be more effective and responsive to the complexities of routine care.

## Supplementary information

Supplementary information

## Data Availability

No datasets were generated or analysed during the current study.
