# Supplementary material for: The present and future of blended care: current research and introduction to the B-FIT framework
Source: NPJ Digit Med. 2026 Mar 26;9:436. doi: 10.1038/s41746-026-02526-4 (PMC13249857; doi:10.1038/s41746-026-02526-4)
Supplement: Supplementary file 1 — Supplementary information [file 41746_2026_2526_MOESM1_ESM.docx]

**Supplementary Note 1: An Overview of Current Literature (Randomized Controlled Trials only) on BC Presented in the Main Text, Aligned With the Dimensions of the B-FIT Framework.**

**Table 1.** Applying B-FIT to an Excerpt of Existing BC Literature (Randomized Controlled Trials)

| **Study** | **Sample** | **Intended Focus (B-FIT)** | **Intended Integration (B-FIT)** | **Intended Timing (B-FIT)** | **Sample Size per Group** | **Main Findings** |
| --- | --- | --- | --- | --- | --- | --- |
| Berger et al. (2018) | Adults with depression in outpatient psychotherapy | F2F-dominant | Low–moderate | During F2F treatment | Intervention (n = 51); Treatment-as-usual control (n = 47) | Adjunct digital intervention improved depressive symptoms more than psychotherapy alone. Primary outcome measure: Beck Depression Inventory–II (BDI-II) at 12 weeks. |
| Bisson et al. (2022) | Adults with mild / moderate post-traumatic stress disorder | Digital-dominant | Moderate | During F2F treatment | Guided internet-based cognitive behavioral therapy–trauma-focused (iCBT-TF; n = 97); Face-to-face cognitive behavioral therapy–trauma-focused (CBT-TF; n = 99) | Guided iCBT-TF was non-inferior to F2F CBT-TF. Primary outcome measure: Clinician-Administered PTSD Scale for DSM-5 (CAPS-5) at 16 weeks. |
| Carroll et al. (2008) | Adults seeking treatment for substance dependence (outpatient) | F2F-dominant^1^ | Low | During F2F treatment | Treatment as usual (n = 38); Computer-based training in CBT (CBT4CBT; n = 39) | CBT4CBT participants submitted more urine specimens negative for any type of drugs and tended to have longer continuous periods of abstinence. Primary outcomes: urine toxicology screens and frequency of substance use. |
| Kemmeren et al. (2023) | Adults with depression in specialized care | Balanced | High | During F2F treatment | Blended CBT (n = 53); Treatment as usual (n = 50) | Depressive symptoms declined in both groups. Primary outcome measure: Patient Health Questionnaire-9 (PHQ-9) from baseline to T3. |
| Krämer et al. (2021) | Adults with depression waiting for outpatient psychotherapy | Digital-dominant | Low | Before F2F treatment | Intervention (n = 67); Control (n = 69) | Web-based intervention + treatment as usual reduced depressive symptoms more than treatment as usual. Primary outcome measure: Center for Epidemiologic Studies Depression Scale (CES-D). |
| Ly et al. (2015) | Adults with major depression recruited by self-referral | Digital-dominant^2^ | Moderate–high | During F2F treatment | Blended treatment (n = 46); Full behavioral activation (n = 47) | No differences between groups. Primary outcome measure: Beck Depression Inventory–II (BDI-II) assessed at pre-, post-, and 6-month follow-up. |
| Mathiasen et al. (2022) | Adults with major depression | Balanced | High | During F2F treatment | Blended CBT (n = 38); Face-to-face CBT (n = 38) | No differences between groups at 6-month follow-up. Primary outcome: Patient Health Questionnaire-9 (PHQ-9). |
| Romijn et al. (2021) | Adults with anxiety disorders | Balanced | Moderate | During F2F treatment | Blended CBT (n = 52); Face-to-face CBT (n = 62) | No between-group differences in the primary outcome. Primary outcome: Beck Anxiety Inventory (BAI). |
| Schaeuffele et al. (2025) | Adults in outpatient psychotherapy | Balanced | High | During F2F treatment | Blended care (n = 583); Psychotherapy only (n = 576) | No differences in primary or secondary outcomes between groups. Primary outcome mental distress (composite PHQ-8 and GAD-7) |
| Sethi (2013) | Young adults with generalized anxiety or depression | Balanced | Not reported | During F2F treatment | Face-to-face CBT (n = 21); Computerized CBT (n = 23); Combined CBT (n = 22); Control (n = 23) | MoodGYM plus face-to-face CBT was more effective than single-modality conditions. Primary outcomes: Kessler Psychological Distress Scale (K10) and Depression Anxiety Stress Scales-21 (DASS-21). |
| Thase et al. (2018) | Adults with major depressive disorder | F2F-dominant | High | During F2F treatment | Computer-assisted CBT (n = 77); Standard CBT (n = 77) | Blended CBT yielded similar outcomes to standard CBT. Primary outcome: Hamilton Rating Scale for Depression, 17-item version (HAM-D-17) at week 16. |
| Witlox et al. (2021) | Older adults with mild to moderately severe anxiety symptoms, recruited from general practices | Digital-dominant | High | During F2F treatment | Blended acceptance and commitment therapy (n = 157); Face-to-face CBT (n = 157) | No group differences. Primary outcome measure: Generalized Anxiety Disorder-7 (GAD-7). |
| Zwerenz et al. (2017) | Depressed inpatients | F2F-dominant | Low | During F2F treatment | Online self-help + inpatient psychotherapy (n = 115); Active control + inpatient psychotherapy (n = 114) | Depressive symptoms were significantly lower in the intervention group compared to the active control at the end of the intervention. Primary outcome measure: Beck Depression Inventory–II (BDI-II) at end of the intervention. |

Note. Randomized Controlled Trials (RCTs) on blended care (BC) were mapped onto the three dimensions of the B-FIT framework. Although B-FIT is conceptualized as a dimensional model, we applied simple, surface-level categories to illustrate how existing studies vary along each dimension. For Focus, we distinguished whether the blend was intended to contain more face-to-face (F2F-dominant) or more digital (digital-dominant) content. Integration was coded as low, moderate, or high, depending on how interwoven the digital and in-person components were described. Timing was classified as before, during, or after face-to-face therapy. We emphasize that this represents a surface-level mapping of existing work; more granular or formalized operationalizations (e.g., checklists, rating tools, or measurement guidelines) represent important future directions for developing B-FIT in more detail.

1 Six sessions of a digital CBT program were combined with weekly "general drug counselling" and F2F group sessions. Weekly counseling is not described, so we based the decision for classification on the frequency: As the frequency of the F2F components appears higher, we classed this intervention as "F2F dominant".

2 Four F2F sessions were combined with weekly homework with an app over the course of nine weeks. As the app took over the Behavioral Activation component and was used more frequently than the F2F sessions, we classed this intervention as "digital dominant".

**Supplementary Note 2: Example of a Clinical Application of the B-FIT Framework**

A brief clinical vignette can illustrate how B-FIT’s three core dimensions can support therapy planning.

**Ms. K., 34 years old**, seeks treatment for extensive worries, recurring sleep difficulties, and ongoing conflicts with her partner. Because her work schedule varies from week to week, she and her therapist decide to engage in BC. B-FIT helps them jointly clarify how BC can be implemented for her.

**Focus:**  Given Ms. K’s complex, transdiagnostic symptoms, they opt for the modular, transdiagnostic online tool TONI (Behr et al., 2024), which allows them to select personalized content for her to work on between face-to-face sessions.The therapist chooses to have a balanced **focus** between digital and face-to-face components. In therapy sessions, the emphasis is on introducing new concepts and conducting experiential exercises; the digital intervention deepens and consolidates these concepts. For some issues, e.g., her sleep difficulties, the digital part is used in a self-guided manner by working through TONI’s sleep module and using sleep-tracking tools. In this way, each therapeutic task is assigned to the format that best suits its complexity and communication demands.

**Integration:** The therapist and patient decide on a high degree of integration. In this case, skill practice and symptom monitoring need to feed directly into the next session, and a tightly integrated blend ensures that digital activities naturally flow into the in-person work. This reduces fragmentation, keeps Ms. K.’s workload organized, and allows the therapist to respond to digital data as soon as it becomes relevant. For clients who can feel overwhelmed by managing multiple tools independently, or who benefit from a clear, structured treatment pathway, high integration may increase engagement, continuity, and fidelity to the therapeutic plan. In practice, this means that Ms. K.’s digital sleep logs and exercises in TONI are not treated as optional or separate “homework,” but as an active part of therapy. Because TONI allows the therapist to view her entries and track progress, they routinely review the digital work together during the session, sometimes opening the tool jointly. Mrs. K. can also send messages to the therapist through TONI between sessions. This close weaving of online and face-to-face elements helps Ms. K. experience treatment as one coherent process rather than a fragmented split between “online work” and “therapy.”

**Timing:** For Ms. K., the therapist recommends placing the digital components *during* the active course of therapy, with a particular emphasis on using them *between individual sessions*. This timing allows TONI to support the therapeutic work exactly when Ms. K. is trying to apply skills in her daily life. Between-session homework may be an active ingredient of treatment (see main manuscript). By positioning TONI’s digital tasks between sessions, the therapist ensures that Ms. K. receives structure and guidance at the moments when she is most likely to need it. This timing supports the enactment of strategies in real-world contexts and strengthens the link between in-session learning and everyday application.

**Supplementary Note 3: Lessons learned from implementation**

To examine lessons learned from implementation efforts on BC, BC experts were recruited via snowball-system from January to February 2025. This approach was specifically chosen to gather insights from individuals who possess practical experience in the development, implementation, and evaluation of blended care projects, ensuring that the perspectives reflected in our qualitative data are grounded in real-world application. Participants answered open-ended questions regarding their experiences with the development phase of blended care projects and their main learnings derived from the evaluation and implementation, whereby only the latter was analysed. A total of nine participants from six BC projects and one planned project responded to the survey request. Authors of this manuscript were also amongst the surveyed experts.  Despite the small sample size of nine, we strived for an explorative thematic overview by engaging a diverse group of experts. The qualitative data obtained from the email responses were analyzed using MAXQDA (version 24.3.0) through a thematic analysis approach (Braun & Clarke, 2006). Thematic analysis was conducted using a systematic approach based on the six phases outlined by Braun & Clarke (2006). We began by familiarizing ourselves with the qualitative data, followed by generating initial codes based on three predefined categories (Learnings Implementation, Learnings Evaluation, and Learnings Development), while remaining open to emerging codes leading to a comprehensive codesystem. Subsequent steps involved grouping these codes into potential themes, rigorously reviewing and refining them, and finally defining and naming the themes to ensure clarity and relevance. The end result highlighted the practical implications drawn from the data, providing actionable insights into the successful implementation of blended care methodologies.

Alongside the findings presented in Figure 3 of the manuscript, findings from the qualitative analysis are summarized here. Participants emphasized that effective implementation necessitates meticulous planning from the project proposal stage: According to their perspective, this needs to encompass both temporal and monetary considerations to ensure adequate practitioner training and remuneration, alongside strategies for post-project continuation, such as server costs or device provision (#2,4,8). Given the substantial implementation costs, one participant highlighted that exploring health insurance coverage for face-to-face sessions is advisable (#8). One participant shared that involving health insurance providers (#4) presents a potentially effective avenue for implementation, while engaging practitioners or clinics may prove challenging due to perceived time constraints and inadequate compensation for training (#7). One participant experienced that implementation in outpatient settings resulted in higher adherence compared to inpatient environments, where the human factor (e.g. engagement of staff) plays a critical role (#3). Consistent communication between the research teams and practitioners was seen as essential to maintain the practitioners’ commitment to the study/intervention by one participant (#6). This is complemented by another perspective that highlighted that practitioner engagement within the study was perceived much lower than the (previously) reported need (#7).  Comprehensive planning of practitioner training, incorporating technical aspects and client onboarding, is imperative, with technical support being paramount (#5,6,8,2). Recognising the diverse patient needs for personal contact, the utilisation of flexible blended care settings may enhance engagement according to one participant (#5).

**Supplementary Table 2: Participating projects in the implementation survey.**

| **Project** | **Type of intervention** | **Indication** | **Information** |
| --- | --- | --- | --- |
| No title | n/a | Postpartal depression | -.- |
| E-COMPARED | App & face-to-face; therapy | Depression | https://klips.phil.fau.de/ecompared  https://www.e-compared.eu/ |
| Moodbuster | App & face-to-face; therapy | Depression | https://www.moodbuster.science/de/ |
| Gesund und Glücklich Aufwachsen (GuG-Auf) and Gesund und Glücklich Aufwachsen Online (GuG-Auf-Online) | Group-based prevention program supported with an app | Parents with experience of depression and their children (8 - 17 years) | https://prodo-group.com/angebote-fuer-familien/gug-auf-gesund-und-gluecklich-aufwachsen/ |
| PSYCHOnlineTHERAPIE  (*n*=2) | Behavioural therapy-based online lessons presented via browser- or app-based platform; embedded in outpatient psychotherapy | Patients with depression and anxiety disorders undergoing outpatient psychotherapeutic treatment | https://psychonlinetherapie.de/ |
| REMOTION | Web-based; various settings as an adjunct to therapy | Patients with different disorders (transdiagnostic) | .-. |
| I-PREGNO  (*n*=2) | App + Counseling | Psychosocially stressed parents from pregnancy to 1 year after birth | https://www.i-pregno.org/ |

Note. All projects/interventions were in German language. Two projects were the subject of two responses each, with both responses being given full consideration on account of the differing perspectives they offer.

**References in Supplementary Material:**

Behr, S., et al. TONI - One for all? Participatory development of a transtheoretic and transdiagnostic online intervention for blended care. *Internet Interventions* 35, 100723 (2024).

Berger, T., Krieger, T., Sude, K., Meyer, B., & Maercker, A. Evaluating an e-mental health program (“deprexis”) as adjunctive treatment tool in psychotherapy for depression: Results of a pragmatic randomized controlled trial. *Journal of Affective Disorders* 227, 455–462 (2018).

Bisson, J. I., et al. Guided internet-based cognitive behavioural therapy for post-traumatic stress disorder: Pragmatic, multicentre, randomised controlled non-inferiority trial (RAPID). *BMJ* 377, e070072 (2022).

Carroll, K. et al. Computer-assisted delivery of cognitive-behavioral therapy for addiction: A randomized trial of CBT4CBT. *American Journal of Psychiatry* 165(7), 881–888 (2008).

Braun, V., & Clarke, V. Using thematic analysis in psychology. *Qualitative Research in Psychology* 3(2), 77–101 (2006).

Kemmeren, L. L., et al. Effectiveness of blended cognitive behavioral therapy versus treatment as usual for depression in routine specialized mental healthcare: E-COMPARED trial in the Netherlands. *Cognitive Therapy and Research* 47(3), 386–398 (2023).

Krämer, L. V., Helbig, S., Maercker, A., & Bockting, C. L. Effectiveness of a guided web-based intervention to reduce depressive symptoms before outpatient psychotherapy: A pragmatic randomized controlled trial. *Psychotherapy and Psychosomatics* (2021).

Ly, K. H. et al. Smartphone-supported versus full behavioural activation for depression: A randomised controlled trial. *Behaviour Research and Therapy* 72, 61–70 (2015).

Mathiasen, K. et al. Clinical effectiveness of blended cognitive behavioral therapy compared with face-to-face cognitive behavioral therapy for adult depression: Randomized controlled non-inferiority trial. *Journal of Medical Internet Research* 24(9), e36577 (2022).

Romijn, G. et al. Effectiveness of blended cognitive behavioral therapy for anxiety disorders in specialized care: A randomized controlled trial. *Journal of Anxiety Disorders* 77, 102338 (2021).

Schaeuffele, C. et al. (2026). Increasing the effectiveness of psychotherapy in routine care through transdiagnostic online modules? Randomized controlled trial investigating blended care. *Journal of Consulting and Clinical Psychology*, *94*(1), 11–25.

Sethi, S. Treating youth depression and anxiety: A randomised controlled trial examining the efficacy of computerised versus face-to-face cognitive behaviour therapy. *Australian Psychologist* 48(4), 249–257 (2013).

Thase, M. E. et al. Improving the efficiency of psychotherapy for depression: Computer-assisted versus standard cognitive-behavioral therapy. *American Journal of Psychiatry* 175(3), 242–250 (2018).

Witlox, M. et al. Blended acceptance and commitment therapy versus standard cognitive-behavioral therapy for older adults with anxiety in primary care: A randomized controlled trial. *Journal of Anxiety Disorders* 78, 102354 (2021).

Zwerenz, R. et al. Online self-help as an add-on to inpatient psychotherapy: Efficacy of a new blended treatment approach. *Psychotherapy and Psychosomatics* 86(6), 341–351 (2017).
